# Association between the triglyceride glucose index and heart failure: NHANES 2007–2018

**DOI:** 10.3389/fendo.2023.1322445

**Published:** 2024-01-22

**Authors:** Fudan Zhang, Xu Hou

**Affiliations:** ^1^ Department of Endocrinology, Shandong Provincial Hospital Affiliated to Shandong First Medical University, Jinan, China; ^2^ Department of Endocrinology and Metabolic Diseases, Shandong Provincial Hospital Affiliated to Shandong First Medical University, Jinan, China

**Keywords:** triglyceride glucose index, heart failure, NHANES, nonlinear associations, cardiovascular disease

## Abstract

**Background:**

Patients with heart failure (HF) were compared with non-HF people to explore the relationship between the triglyceride glucose (TyG) index and HF in participants with cardiovascular and cerebrovascular diseases.

**Methods:**

TyG index was calculated as ln [fasting triglyceride (mg/dL) × fasting glucose (mg/dL)/2]. Multivariate logistic regression models were used to investigate the association between the TyG index and the risk of HF. Restricted cubic spline (RCS) analysis was applied to evaluate the dose–response relationship between the TyG index and the risk of HF.

**Results:**

National Health and Nutrition Examination Survey (NHANES) (2007–2018) was used to analyze the association between TyG and HF in patients. A total of 13,825 participants who had their TyG index measured were included, involving 435 individuals with HF and 13,390 individuals without HF. Those with HF had higher levels of the TyG index compared with those without HF (8.91 ± 0.74 vs. 8.57 ± 0.66, *p* < 0.001). The odds ratio (OR) of HF for the TyG index from logistic regression was 1.644 and 1.057 in Model 1 (without adjusting for any variables) and Model 4 (adjusted for all covariates), respectively. Compared with individuals with Q1, a higher TyG index was related to the increased risk of HF. Model 1 showed that there was a linear dose–response relationship between the TyG index and HF (*p* = 0.686). The TyG index predicted the area of the receiver operating characteristic (ROC) curve of 0.602 (95% CI: 0.575–0.629, *p* < 0.001) and the optimal cutoff value was 8.91.

**Conclusion:**

The TyG index was positively associated with the risk of HF. The TyG index may be a therapeutic target and an important predictor of HF.

## 1 Introduction

Heart failure (HF) is a complex clinical syndrome caused by various cardiovascular diseases and is characterized by high morbidity and mortality worldwide (1). The progress of social population aging and the increase of chronic basic diseases such as coronary heart disease and hypertension will all lead to the increased incidence of HF in China (2). Thus, how to early identify and diagnose individuals with a high risk of HF is of crucial importance. HF mainly presents with a series of clinical syndromes such as dyspnea, cough, sputum, fatigue, palpitations, jugular vein irritability, lower limb edema, and anuria (1), which is the terminal stage of a variety of cardiovascular diseases and one of the main causes of death and disability. Many clinical classification systems have been used for HF, including those based on symptom severity or on disease progression. Nowadays, it is mainly classified by anatomy, left ventricular ejection fraction, and course of disease. The HF was classified by left HF, right HF, and whole HF. There are many factors (3) found that can cause HF, including coronary atherosclerotic heart disease (coronary heart disease), hypertension, heart valvular disease, and metabolic diseases.

The TyG index is a marker used to assess insulin resistance (IR) (4–6). IR often coexists with the risk factors for common cardiovascular diseases such as hyperlipidemia, obesity, atherosclerosis, and coagulation disorders. The cardiovascular system is an important target organ of insulin action. IR can change the cardiac metabolism by increasing myocardial oxygen consumption, and then cause diastolic dysfunction, activate the renin angiotensin aldosterone system, increase water and sodium retention, accelerate ventricular remodeling, and lead to the occurrence of cardiac insufficiency and cardiomyopathy (7). Some research (8) found that IR assessed by the TyG index had high sensitivity (96.5%) and specificity (85.0%) compared with the gold standard high insulin-positive glucose clamp method. Increasing research evidence indicates that the TyG index is the influencing factor for the occurrence and development of HF (9–11). Recently, several studies have reported (12) TyG, the correlation between index and HF. A study of the TyG index and HF risk in the US community population found that each standard deviation increase in the TyG index caused a corresponding 15% increase in HF risk. Another risk assessment of 922 patients after percutaneous coronary intervention found a clear increasing trend in the incidence of HF with increasing TyG index. Furthermore, the study by Han et al. found that TyG was associated with the occurrence of cardiovascular death or HF readmission in patients with HF.

Based on the above background, this study aimed to evaluate the cross-sectional association between TyG and HF with data from the NHANES.

## 2 Materials and methods

### 2.1 Study population

**Figure 1:**
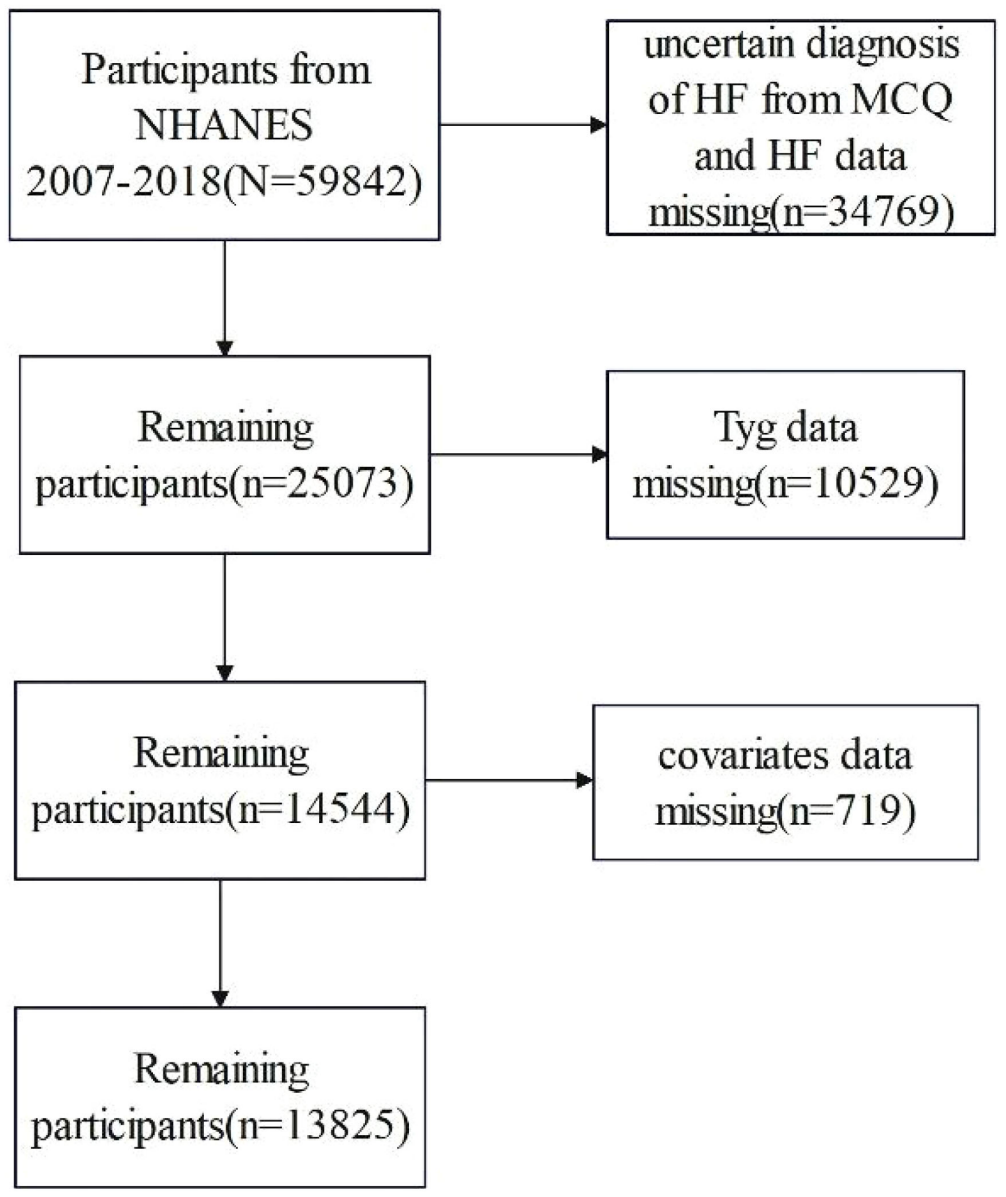
The flowchart of selection of included studies.

The study subjects were female and male participants drawn from the NHANES (13). This study incorporates the populations in the NHANES database from 2007 to 2008, 2009 to 2010, 2011 to 2012, 2013 to 2014, 2015 to 2016, and 2017 to 2018. The NHANES database was a nationwide and ongoing cross-sectional survey conducted among the non-institutionalized US population. The database is publicly available at https://www.cdc.gov/nches/nhanes. The NHANES protocol was approved by the National Center for Health Statistics (NCHS) Research Ethics Review Board. 2007 to 2008, 2009 to 2010, 2011 to 2012, 2013 to 2014, 2015 to 2016, and 2017 to 2018 NHANES cycles were selected, and a total of 59,482 participants were included in the study. We excluded participants with missing HF, TyG index, and covariates. We also excluded uncertain diagnosis of HF. Finally, a total of 13,825 participants were included in the present study. The flowchart of sample selection is shown in **Figure 1**.

We used the “MCQ160B” variable from MCQ in the questionnaire to diagnose HF, which asked “Someone ever told you had congestive HF?” Some previous NHANES-based articles have been published (14–16). The demographic information and characteristics, which consist of age, gender, body mass index (BMI), race, education, and income, were obtained by interviewing. The TyG index (17) was calculated by TyG = Ln [fasting TG (mg/dL) ×fasting glucose (mg/dL)/2]. Thus, we included triglyceride and fasting glucose data to calculate the TyG index. The TyG index was divided into four groups, according to the quartiles (Q1: <P25, Q2: P25–P50, Q3: P50–P75, and Q4: P75). Homeostasis Model Assessment of Insulin Resistance (HOMA-IR) (18) was calculated by HOMA-IR = (fasting glucose (mmol/L)×fasting insulin (μU/ml)/22.5).

### 2.2 Covariates

Covariates included demographic data including age, race, gender, education, and household income level. Personal data included BMI, sex, and age. Age was divided into three groups (20 to 40, 40 to 60, and ≥61), family income level was divided into two groups according to the score of income poverty (low-income families and high-income families), and BMI was divided into three groups (<25.0, 25.0–29.9, and ≥30.0 kg/m^2^). Education level was divided into three groups based on high school (less than high school, high school, and more than high school), and race was divided into five groups (Mexican, Hispanic, white, black, and others). Individuals were considered to have coronary heart disease if they answered yes to the question “Someone ever told you had coronary heart disease?” Individuals were considered to have angina pectoris if they answered yes to the question “Someone ever told you had angina pectoris?” Individuals were considered to have heart attack if they answered yes to the question “Someone ever told you had heart attack?” Individuals were considered to have stroke if they answered yes to the question “Someone ever told you had stroked?” Type 2 diabetes mellitus (19) was defined as a diagnosed case of diabetes mellitus with insulin or oral hypoglycemic agents and fasting glucose levels above 7.0 mmol/L (126 mg/dL) or glycated hemoglobin A1c (HbA1c) levels above 6.5%.

### 2.3 Statistical analysis

All statistical analyses were performed using the R software (R version 4.3.1) and SPSS (25.0). NHANES collected data nationally representative through a complex sampling design and the use of sample weights. In this paper, the data are weighted according to the sample weight calculation method recommended by NHANES. The demographic characteristics of the HF and non-HF groups were described by the composition ratio (%), and the weighted χ^2^ test was used to compare the differences between the two groups. Weighted univariate logistic regression analysis calculated the independent risk factors for HF. The TyG index was divided into Q1 to Q4 groups according to quartiles, and the Q1 group was used as the reference group to adjust for potential confounders, including age, sex, BMI, poverty income ratio, education, coronary heart disease, heart attack, stroke, hypertension, and diabetes. A multivariate logistic regression model was used to analyze the relationship between the TyG index and HF risk, and the results were reported as the OR and its 95% confidence interval (95% CI) and *p*-values. No covariates were adjusted for as indicated in Model 1. Age, sex, and BMI were adjusted in Model 2; coronary heart disease, angina pectoris, heart attack, stroke, and hypertension were adjusted in Model 3. Model 4 was adjusted for all covariates, including age, sex, BMI, coronary heart disease, angina pectoris, heart attack, stroke, hypertension, and diabetes. In addition, we carried out several restricted cubic spline (RCS) analyses to explore the non-linear dose–response relationship between HF and the TyG index in the whole population, and four knots were placed at the 5th, 35th, 65th, and 95th percentiles. ROC (20) curves were used to evaluate the predictive efficacy of TyG on patients with HF. 1 − Specificity is the *X* axis in the ROC curve plot, and the *Y* axis represents the sensitivity. The accuracy of index in predicting the HF determines the offline area (AUC) of the ROC curve. *p*-values <0.05 were considered as statistically significant differences in chi-square test, ANOVA, RCS analyses, and ROC curves.

## 3 Results

### 3.1 Baseline characteristics

A total of 13,825 study subjects, namely, 6,651 men and 7,174 women, were included in this study, as shown in **Figure 1**. Of the 13,825 subjects, 435 were diagnosed with HF, representing 3.14% of the total population. Compared with non-HF people, the included HF population group had lower education level and income level and higher body mass index (*p* < 0.001). Male, age, coronary heart disease, heart attack, hypertension, angina pectoris, and stroke were higher among the HF population (*p* < 0.001). In addition, diabetes incidence was higher among the HF population (**Table 1**). In terms of laboratory indicators, HF patients had higher levels of triglyceride and fasting plasma glucose. Notably, the HF group exhibited a significantly higher TyG index and HOMA-IR than the non-HF group (8.91 vs. 8.57, *p* < 0.001 for TyG index; 7.27 vs. 4.01, *p* < 0.001 for HOMA-IR).

**Table 1:** Baseline characteristics of the HF and non-HF groups.

Variables	Heart failure	Non-heart failure	Overall	*p*
Count	435 (3.1)	13,390 (96.9)	13,825	
Age, mean (SE)	66.12 (12.88)	47.35 (16.63)	47.81 (16.80)	<0.001
BMI, mean (SE)	31.56 (7.97)	29.01 (6.84)	29.07 (6.88)	<0.001
≤24.9, *n* (%)	94 (21.6)	3,971 (29.7)	4,065	
25–29	117 (26.9)	4,450 (33.2)	4,567	
≥30	224 (51.5)	4,969 (37.1)	5,193	
Sex, *n* (%)				<0.001
Male	222 (51.1)	6,429 (48.1)	6,651 (48.1)	
Female	213 (48.9)	6,961 (51.9)	7,174 (51.9)	
TyG, mean (SE)	8.91 (0.74)	8.57 (0.66)	8.58 (0.66)	<0.001
Q1	62 (14.2)	3,393 (25.3)	3,455 (25.00)	
Q2	100 (22.98)	3,360 (25.1)	3,460 (25.02)	
Q3	105 (24.13)	3,348 (25.01)	3,453 (24.97)	
Q4	168 (38.6)	3,289 (24.6)	3,457 (25.01)	
Triglyceride, mg/dl, mean (SE)	146.34 (153.4)	124.3 (107.7)	125.1 (109.5)	<0.001
FPG, mg/dL, mean (SE)	126.22 (49.76)	109.46 (35.67)	109.98 (36.31)	<0.001
HOMAIR, mean (SE)	7.27 (15.54)	4.01 (6.80)	4.11 (7.26)	<0.001
Income, *n* (%)				<0.001
Low	299 (68.8)	6,623 (49.5)	6,922 (50.1)	
High	136 (31.2)	6,767 (50.5)	6,903 (49.9)	
Education, *n* (%)				<0.001
Less than high school	152 (34.9)	3,205 (23.9)	3,357 (24.3)	
High school	110 (25.3)	3,014 (22.5)	3,124 (22.6)	
More than high school	173 (39.8)	7,171 (53.6)	7,344 (53.1)	
CAD, *n* (%)				<0.001
Yes	171 (39.3)	399 (2.9)	570 (4.1)	
No	264 (60.7)	12,991 (97.1)	13,255 (95.9)	
Angina, *n* (%)				<0.001
Yes	97 (22.3)	247 (1.8)	344 (2.5)	
No	338 (77.7)	13,143 (98.2)	13,481 (97.5)	
Heart attack, *n* (%)				<0.001
Yes	188 (43.2)	399	587 (4.2)	
No	247 (56.8)	12,991	13,238 (95.8)	
Hypertension, *n* (%)				<0.001
Yes	352 (80.9)	4,733 (35.3)	2,085 (15.1)	
No	83 (19.1)	8,657 (64.7)	8,740 (84.9)	
Stroke, *n* (%)				<0.001
Yes	91 (20.9)	423 (3.2)	514 (3.7)	
No	344 (79.1)	12,967 (96.8)	13,311 (96.3)	
Diabetes, *n* (%)				<0.001
Yes	216 (49.7)	2,432 (18.2)	2,648 (19.2)	
No	211 (48.5)	10,721 (80.1)	10,932 (79.1)	

To further investigate the correlation between the TyG index and HF, **Table 2** presents the baseline characteristics of the patients divided into quartiles based on the TyG index (**Table 2**). The TyG index was divided into four groups, namely, ≤8.15, 8.16–8.57, 8.58–9.03, and ≥9.03 in Q1, Q2, Q3, and Q4, respectively. Individuals in Q4 had a higher BMI, with lower education, lower household income level, and more male population. The prevalence of hypertension, coronary artery disease, heart attack, stroke, and angina pectoris had a significant difference (*p* < 0.001) among Q1, Q2, Q3, and Q4, while Q2 occupied the highest proportion of angina pectoris (18.2%). Individuals in Q2 had a higher risk of stroke than individuals in Q3. Among the patients in the Q4 group, they exhibited a higher incidence of diabetes. With the increase of the TyG index, there was a gradual increase in the incidence of diabetes.

**Table 2:** Baseline characteristics according to TyG index quartiles.

Variable	Q1	Q2	Q3	Q4	Overall	*p*
Count	3,455	3,460	3,453	3,457	13,825	
Sex, *n* (%)						<0.001
Male	1,296 (37.5)	1,642 (47.5)	1,768 (51.2)	1,945 (56.3)	6,651 (48.1)	
Female	2,159 (62.5)	1,818 (52.5)	1,685 (48.8)	1,512 (43.7)	7,174 (51.8)	
BMI, kg/m², *n* (%)						<0.001
≤24.9	1,650 (47.8)	1,157 (33.4)	788 (22.8)	470 (13.6)	4,065 (29.4)	
25–29	972 (28.1)	1,204 (34.8)	1,194 (34.6)	1,197 (34.6)	4,567 (33.1)	
≥30	833 (24.1)	1,099 (31.8)	1,471 (42.6)	1,790 (51.8)	5,193 (37.5)	
Age, years, *n* (%)						<0.001
≤40	1,758 (50.1)	1,210 (34.9)	1,014 (29.4)	813 (23.5)	4,795 (34.6)	
41–60	996 (28.8)	1,148 (33.2)	1,207 (34.9)	1,351 (39.1)	4,702 (34.1)	
≥61	701 (21.1)	1,102 (31.9)	1,232 (35.7)	1,293 (37.4)	4,328 (31.3)	
Triglyceride, mg/dL, mean (SE)	53.9 (13.7)	87.1 (14.2)	124.8 (23.3)	234.2 (169.4)	125.1 (109.6)	<0.001
FPG, mg/dL, mean (SE)	94.4 (11.2)	100.8 (14.4)	107.7 (20.8)	136.4 (59.1)	109.9 (36.6)	<0.001
HOMAIR, mean (SE)	2.1 (1.8)	2.8 (3.5)	4.1 (4.7)	7.4 (12.4)	4.1 (7.3)	<0.001
Race, *n* (%)						<0.001
Mexican	351 (10.2)	452 (13.1)	595 (17.2)	687 (19.9)	2,085 (15.1)	
Hispanic	275 (7.9)	334 (9.7)	415 (12.1)	405 (11.7)	1,429 (10.3)	
White	1,304 (37.7)	1,507 (43.5)	1,498 (43.4)	1,583 (45.8)	5,892 (42.6)	
Black	1,070 (30.9)	756 (21.8)	517 (14.9)	403 (11.7)	2,746 (19.8)	
Other	455 (13.3)	411 (11.9)	428 (12.4)	379 (10.9)	1,673 (12.2)	
Education, *n* (%)						<0.001
Less than high school	604 (17.5)	768 (22.2)	923 (26.7)	1,062 (30.7)	3,357 (24.4)	
High school	715 (20.7)	800 (23.1)	789 (22.8)	820 (23.7)	3,124 (22.5)	
More than high school	2,136 (61.8)	1,892 (54.7)	1,741 (50.5)	1,575 (45.6)	7,344 (53.1)	
Income						<0.001
Low	1,523 (44.1)	1,666 (48.2)	1,823 (52.8)	1,900 (54.9)	6,922 (50.1)	
High	1,922 (55.9)	1,794 (51.8)	1,630 (47.2)	1,557 (45.1)	6,903 (49.9)	
CAD, *n* (%)						
Yes	84 (2.4)	120 (3.5)	148 (4.2)	218 (6.3)	570 (4.2)	
No	3,371 (97.6)	3,340 (96.5)	3,305 (95.8)	3,239 (93.7)	13,255 (95.8)	
Angina, *n* (%)						
Yes	43 (1.2)	63 (18.2)	105 (3.1)	133 (3.8)	344 (2.5)	
No	3,412 (98.8)	3,397 (81.8)	3,348 (96.9)	3,324 (96.2)	13,481 (97.5)	
Hypertension, *n* (%)						<0.001
Yes	828 (23.9)	1,163 (33.6)	1,386 (40.1)	1,708 (49.4)	5,085 (36.8)	
No	2,627 (76.1)	2,297 (66.4)	2,067 (59.9)	1,749 (50.6)	8,740 (63.2)	
Stroke, *n* (%)						<0.001
Yes	93 (2.7)	139 (4.1)	122 (3.5)	160 (4.6)	514 (3.8)	
No	3,362 (97.3)	3,321 (95.9)	3,331 (96.5)	3,297 (95.4)	13,311 (96.2)	
Heart attack, *n* (%)						<0.001
Yes	76 (2.2)	136 (3.9)	169 (4.9)	206 (5.9)	587 (4.3)	
No	3,379 (97.8)	3,324 (96.1)	3,284 (95.1)	3,251 (94.1)	13,238 (95.7)	
Heart failure, *n* (%)						<0.001
Yes	62 (1.8)	100 (2.9)	105 (3.1)	168 (4.8)	435 (3.1)	
No	3,393 (98.2)	3,360 (97.1)	3,348 (96.9)	3,289 (95.2)	13,390 (96.9)	
Diabetes, *n* (%)						<0.001
Yes	188 (7.1)	347 (13.1)	651 (24.6)	1,462 (55.2)	2,648 (19.2)	
No	3,217 (29.4)	3,051 (27.9)	2,728 (25.0)	1,936 (17.7)	10,932 (79.1)	

### 3.2 Association between TyG index and HF

**Figure 2:**
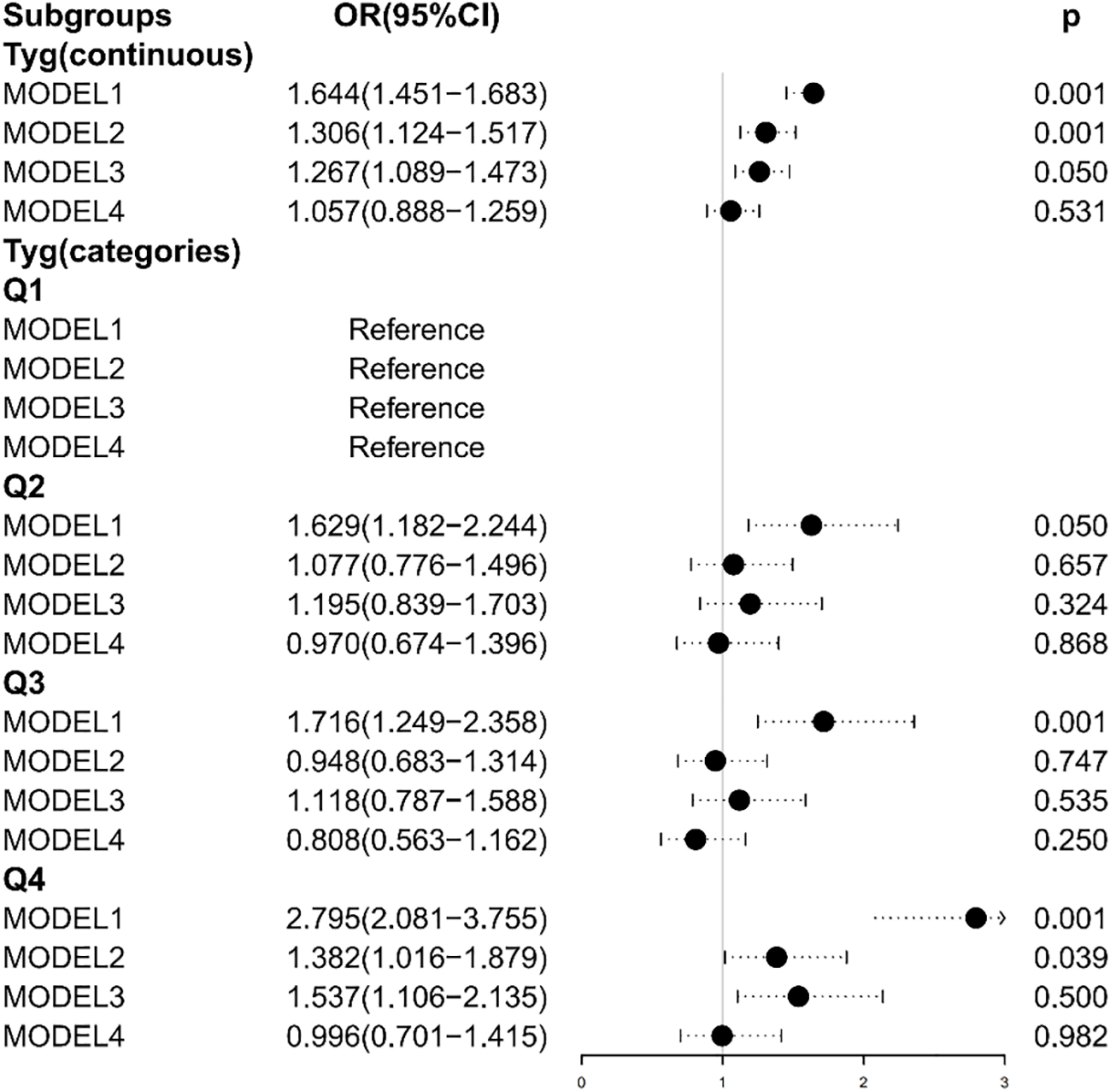
Association between the TyG index and heart failure. Model 1: Unadjusted. Model 2: Adjusted for age, sex, and BMI. Model 3: Adjusted for coronary artery disease, hypertension, heart attack, angina, and stroke. Model 4: Adjusted for age, sex, BMI, coronary artery disease, hypertension, heart attack, angina, stroke, and diabetes.

Multiple logistic regression models study the relationship between TyG index levels and HF. The unadjusted model and the adjusted model are shown in **Table 3** and **Figure 2**. In Model 1, without adjusting for any variables, the OR of HF was increased with a higher TyG index (OR: 1.644, 95% CI 1.451, 1.863, *p* < 0.001), and the test for trend was statistically significant. After adjusting for age, sex, and BMI, the OR in Model 2 was 1.306. Model 3 was adjusted for coronary artery disease, heart attack, stroke, angina pectoris, and hypertension. We found significant correlation between TyG index levels and HF in Model 2 and Model 3. When the models were further adjusted for all covariates, we found no significant correlation between the TyG index and HF (OR [95% CI] = 1.057 [0.888–1.259], *p* = 0.531). In addition, we transformed TyG index into categorical variables (Q1, Q2, Q3, and Q4). Compared with the Q1 group, Q2, Q3, and Q4 in Model 1 had increased risk of HF, and the OR (95% CI) values are 1.629 (1.182–2.244), 1.716 (1.249–2.358), and 2.795 (2.081–3.755), respectively. After adjusting for age, sex, and BMI, the OR in Model 2 was 1.077, 0.948, and 1.382 compared with the Q1 group, respectively. After further adjustment for coronary artery disease, heart attack, stroke, angina pectoris, and hypertension, the OR in Model 3 was 1.195, 1.118, and 1.537 compared with the Q1 group, respectively.

**Table 3:** Logistic regression results showing association between the TyG index and heart failure.

	Model 1	*p*	Model 2	*p*	Model 3	*p*	Model 4	*p*
TyG	OR (95% CI)		OR (95% CI)		OR (95% CI)		OR (95% CI)	
	1.644 (1.451–1.863)	<0.001	1.306 (1.124–1.517)	<0.001	1.267 (1.089–1.473)	<0.05	1.057 (0.888–1.259)	0.531
TyG								
Q1	Ref		Ref		Ref		Ref	
Q2	1.629 (1.182–2.244)	<0.05	1.077 (0.776–1.496)	0.657	1.195 (0.839–1.703)	0.324	0.970 (0.674–1.396)	0.868
Q3	1.716 (1.249–2.358)	<0.001	0.948 (0.683–1.314)	0.747	1.118 (0.787–1.588)	0.535	0.808 (0.563–1.162)	0.250
Q4	2.795 (2.081–3.755)	<0.001	1.382 (1.016–1.879)	0.039	1.537 (1.106–2.135)	<0.05	0.996 (0.701–1.415)	0.982

Model 1: Unadjusted.

Model 2: Adjusted for age, sex, and BMI.

Model 3: Adjusted for coronary artery disease, hypertension, heart attack, angina, and stroke.

Model 4: Adjusted for age, sex, BMI, coronary artery disease, hypertension, heart attack, angina, stroke, and diabetes.

The TyG index levels in Model 2 and Model 3 were positively correlated with HF, while there were no statistically significant differences in the Q2 and Q3 group (Model 2: *p* = 0.657 for the Q2 group, *p* = 0.747 for the Q3 group; Model 3: *p* = 0.324 for the Q2 group, *p* = 0.535 for the Q3 group). After adjusting all covariates, the TyG index was negatively correlated with the risk of HF in Model 4 (OR = 0.970 in the Q2 group; OR = 0.808 in the Q3 group; OR = 0.996 in the Q4 group), while there were no statistically significant differences (*p* = 0.868 for the Q2 group; *p* = 0.250 for the Q3 group; *p* = 0.982 for the Q4 group).

### 3.3 Nonlinear associations and ROC curves

**Figure 3:**
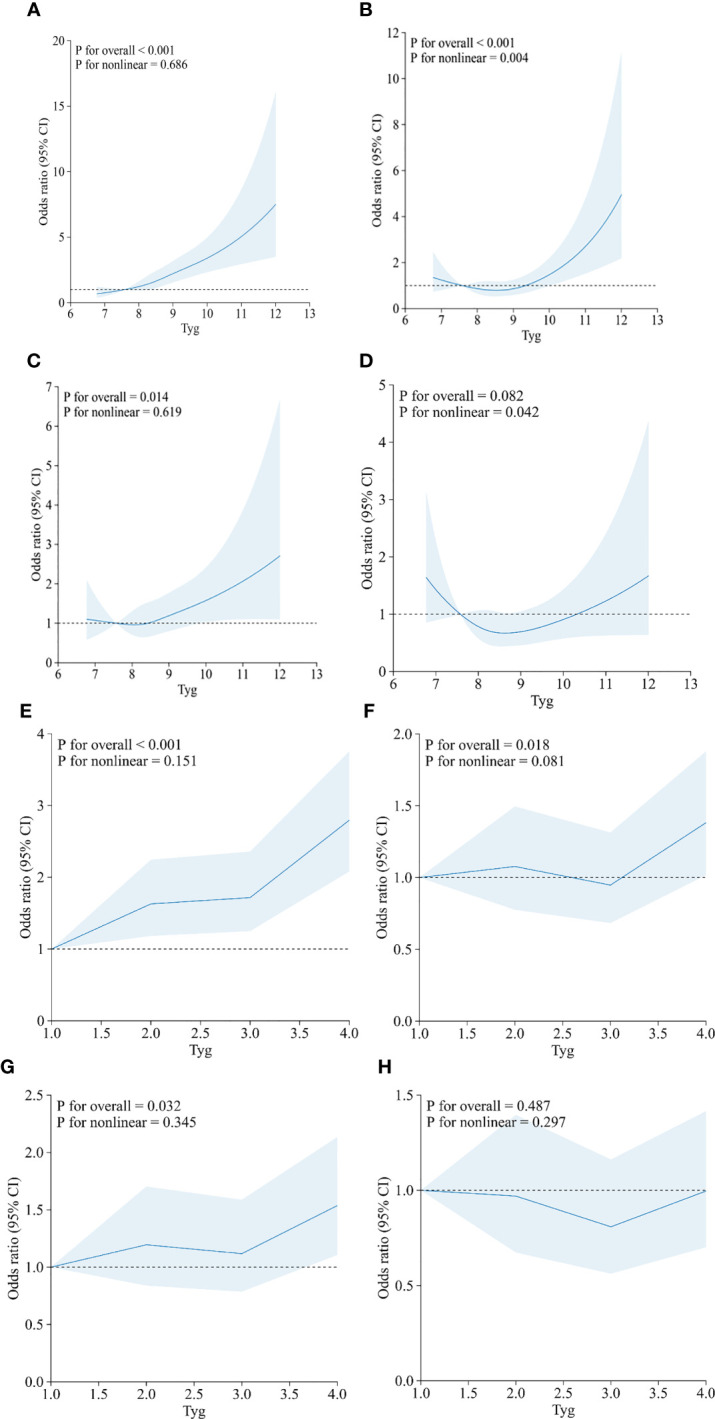
Restricted cubic spline curves between the TyG index (continuous: A–D; categories: E–H) and heart failure. (**A, E**) Model 1 was unadjusted. (**B, F**) Model 2 was adjusted for age, sex, and BMI. (**C, G**) Model 3 was adjusted for coronary artery disease, hypertension, heart attack, angina, and stroke. (**D**, **H**) Model 4 was adjusted for age, sex, BMI, coronary artery disease, hypertension, heart attack, angina, stroke, and diabetes.

The dose–response relationship between the TyG index and HF was further explored using restricted cubic spline plots (**Figure 3**), and results are displayed in **Figure 3**. We built four restricted cubic spline plots according to Model 1, Model 2, Model 3, and Model 4, and all the curves were J-shaped. Model 1 and Model 3 showed that there was a linear dose–response relationship between TyG and HF (*p* = 0.686, *p* = 0.619). However, in Model 2 and Model 4, we found that there was a nonlinear dose–response relationship between TyG and HF (*p* = 0.004, *p* = 0.042). The risk of HF increases with rising TyG index. In addition, we also conducted a restricted cubic spline regression model based on the TyG index quartiles. The results showed that there was a linear dose–response relationship between the TyG index and HF risk in Model 1 (*p* for non-linearity = 0.151), Model 2 (*p* for non-linearity = 0.081), Model 3 (*p* for non-linearity = 0.345), and Model 4 (*p* for non-linearity = 0.297). **Figure S1** showed the relationship between TG, fasting glucose and HF risk.

**Figure 4:**
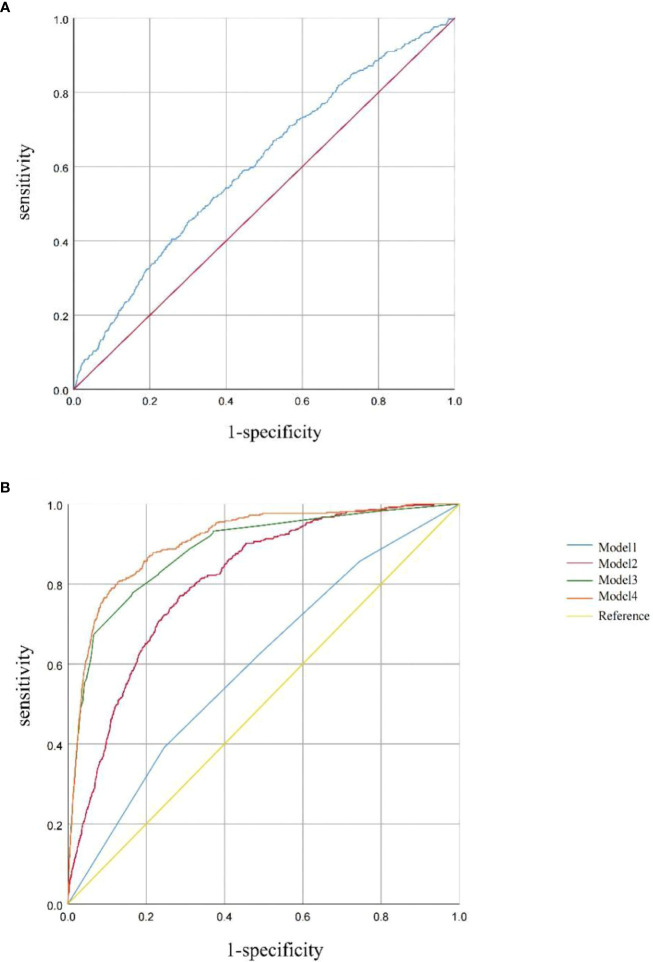
**(A)** ROC curve between the TyG index (continuous) and HF. **(B)** ROC curves between the TyG index (categories) and HF in Model 1, Model 2, Model 3, and Model 4. Model 1 was unadjusted. Model 2 was adjusted for age, sex, and BMI. Model 3 was adjusted for coronary artery disease, hypertension, heart attack, angina, and stroke. Model 4 was adjusted for age, sex, BMI, coronary artery disease, hypertension, heart attack, angina, stroke, and diabetes.

ROC curves were used to evaluate the predictive efficacy of TyG on patients with HF. This area is proportional to the predicted value of the index; the larger the AUC, the higher the predicted value; otherwise, the lower the predicted value is. By drawing the ROC curve, the TyG index (continuous) predicted the area of the ROC curve of 0.602 (95% CI: 0.575–0.629, *p* < 0.001). The optimal cutoff value was 8.91, suggesting some value for predicting the occurrence of HF when the TyG index is greater than 8.91 (**Figure 4**). The corresponding sensitivity and specificity were 45.1% and 70.2%, respectively. Statistical significances were found in predicting the area of the ROC curves in Model 1 (AUC: 0.598, 95% CI: 0.571–0.625, *p* < 0.001), Model 2 (AUC: 0.806, 95% CI: 0.788–0.825, *p* < 0.001), Model 3 (AUC: 0.885, 95% CI: 0.868–0.903, *p* < 0.001), and Model 4 (AUC: 0.908, 95% CI: 0.894–0.923, *p* < 0.001), based on the TyG index quartiles.

## 4 Discussion

This study investigated the relationship between the TyG index and the risk of HF in adults based on data from 2007 to 2018 NHANES. The results showed that the TyG index had a positive relationship with the risk of HF after adjusting for potential confounders. The occurrence of HF increases with the increase of the TyG index. Moreover, there was a significant “J-shaped” dose–response relationship between the TyG index and the risk of HF. When the TyG index exceeded 8.91, the risk of HF increased rapidly with the increase of the TyG index, suggesting that the TyG index may help identify people at high risk of HF. A recent systematic review and meta-analysis (21) for the association between the TyG index and HF showed that a more elevated TyG index was associated with a higher incidence of HF in patients with type 2 diabetes or coronary artery disease. In addition, this meta-analysis found that the TyG index has demonstrated diagnostic ability in distinguishing HF patients from non-HF individuals, which is similar to our results. Our results indicated that the TyG index was positively associated with the risk of HF. The TyG index may be a therapeutic target and an important predictor of HF.

A study of the Mexican population compared the TyG index and the normal glycemic–high insulin clamp test, and concluded a good correlation between the TyG index and the high insulin positive glucose clamp technique; this correlation is similar in gender, obesity, high sensitivity, and specificity for the diagnosis of insulin resistance. IR can cause myocardial energy metabolism disorder, leading to inhibit the use of glucose. Fatty acids become the only energy source. With the degree of IR, the heart decreased the ability to use fatty acids, leading to lipid accumulation and increase. Finally, it will cause cardiac hypertrophy and systolic dysfunction, aggravating the left ventricular remodeling, and leading to the occurrence of HF (22). IR can also impair the AMPK/PGC-1 α signal transduction pathway, which subsequently reduces the mitochondrial membrane potential and ATP synthesis, leading to cardiac remodeling (23). Mitochondrial dysfunction stimulates the increased production of reactive oxygen species (ROS), and, in turn, the increase of ROS levels reduces the capacity of fatty acid oxidation, further driving mitochondrial dysfunction and apoptosis, and leading to lipid accumulation, cardiac fibrosis, and HF. The interaction between ER stress and abnormal calcium processing increases cardiomyocyte apoptosis, necrosis, and autophagy, which, in turn, leads to HF (24). IR can lead to glucose and lipid metabolism disorders, which can induce late advanced glycation product (AGE) through non-enzymatic glycosylation, and AGE can directly promote connective tissue crosslinking, leading to vascular sclerosis. Diabetic patients are often accompanied by systemic macrovascular complications, and the damage to the myocardium can also affect the heart structure and function. IR is a key mechanism in the pathogenesis of type 2 diabetes mellitus (T2DM). IR is an important mechanism for many cardiovascular metabolic abnormalities, especially in T2DM patients, to accelerate the occurrence and progression of cardiovascular lesions. The mechanism of diabetic patients with IR is often attributed to glucose toxicity and lipotoxicity (25). Some studies found that long-term monitoring of TyG index changes was valuable for the early identification of individuals at high risk of diabetes (26, 27).

The TyG index, a composite index composed of the joint participation of fasting blood glucose and serum triglycerides, has the advantages of low economic cost and easy access. This study also has shortcomings. First, this study is based on a cross-sectional design, which can only explore the association between TyG index and HF and cannot verify the causal relationship. The present study only used the baseline TyG index. The cumulative mean of all available TyG indexes from the baseline TyG index to the occurrence of HF or at the end of follow-up can be used as the long-term TyG index to calculate to make the study results more reliable. Secondly, this paper is based on the US NHANES database, which, although with a large enough sample size, is not fully representative of populations around the world. Third, despite adjusting for various confounding factors, other confounds still cannot be excluded.

In the future, more intensive studies are still needed to clarify the role of the TyG index in different cardiovascular diseases and its relationship with the occurrence of cardiovascular adverse events in people with different characteristics. Further validation is needed to benefit patients with cardiovascular disease with the TyG index as a therapeutic target. As a new index to evaluate IR, the TyG index has important clinical significance for the early detection of cardiovascular disease and improving prognosis.

## Data availability statement

The datasets presented in this study can be found in online repositories. The names of the repository/repositories and accession number(s) can be found below: https://www.cdc.gov/nches/nhanes.

## Ethics statement

Ethical approval was not required for the study involving humans in accordance with the local legislation and institutional requirements. Written informed consent to participate in this study was not required from the participants or the participants’ legal guardians/next of kin in accordance with the national legislation and the institutional requirements.

## Author contributions

FZ: Conceptualization, Methodology, Software, Writing – original draft, Writing – review & editing. XH: Funding acquisition, Supervision, Validation, Writing – original draft, Writing – review & editing.

## References

[1] McDonagh TA Metra M Adamo M Gardner RS Baumbach A Böhm M. ESC Guidelines for the diagnosis and treatment of acute and chronic heart failure. Eur Heart J (2021) 42(36):3599–726. doi: 10.1093/eurheartj/ehab368 34447992

[2] McMurray JJ Pfeffer MA. Heart failure. Lancet (2005) 365(9474):1877–89. doi: 10.1016/S0140-6736(05)66621-4 15924986

[3] Saitoh M Dos Santos MR Emami A Ishida J Ebner N Valentova M. Anorexia, functional capacity, and clinical outcome in patients with chronic heart failure: results from the Studies Investigating Co-morbidities Aggravating Heart Failure (SICA-HF). ESC Heart Fail (2017) 4(4):448–57. doi: 10.1002/ehf2.12209 PMC5695184 28960880

[4] Vasques AC Novaes FS de Oliveira Mda S Souza JR Yamanaka A Pareja JC. TyG index performs better than HOMA in a Brazilian population: a hyperglycemic clamp validated study. Diabetes Res Clin Pract (2011) 93(3):e98–e100. doi: 10.1016/j.diabres.2011.05.030 21665314

[5] Guerrero-Romero F Simental-Mendía LE González-Ortiz M Martínez-Abundis E Ramos-Zavala MG Hernández-González SO. The product of triglycerides and glucose, a simple measure of insulin sensitivity. Comparison with the euglycemic-hyperinsulinemic clamp. J Clin Endocrinol Metab (2010) 95(7):3347–51. doi: 10.1210/jc.2010-0288 20484475

[6] Dikaiakou E Vlachopapadopoulou EA Paschou SA Athanasouli F Panagiotopoulos I Kafetzi M. Triglycerides-glucose (TyG) index is a sensitive marker of insulin resistance in Greek children and adolescents. Endocrine (2020) 70(1):58–64. doi: 10.1007/s12020-020-02374-6 32557329

[7] Jeong S Lee JH. The verification of the reliability of a triglyceride-glucose index and its availability as an advanced tool. Metabolomics (2021) 17(11):97. doi: 10.1007/s11306-021-01837-9 34724122

[8] Mazidi M Kengne AP Katsiki N Mikhailidis DP Banach M. Lipid accumulation product and triglycerides/glucose index are useful predictors of insulin resistance. J Diabetes Complications (2018) 32(3):266–70. doi: 10.1016/j.jdiacomp.2017.10.007 29395839

[9] Li X Chan JSK Guan B Peng S Wu X Lu X. Triglyceride-glucose index and the risk of heart failure: Evidence from two large cohorts and a mendelian randomization analysis. Cardiovasc Diabetol (2022) 21(1):229. doi: 10.1186/s12933-022-01658-7 36329456 PMC9635212

[10] Huang H Li Q Liu J Qiao L Chen S Lai W. Association between triglyceride glucose index and worsening heart failure in significant secondary mitral regurgitation following percutaneous coronary intervention. Cardiovasc Diabetol (2022) 21(1):260. doi: 10.1186/s12933-022-01680-9 36443743 PMC9706938

[11] Han S Wang C Tong F Li Y Li Z Sun Z. Triglyceride glucose index and its combination with the Get with the Guidelines-Heart Failure score in predicting the prognosis in patients with heart failure. Front Nutr (2022) 9:950338. doi: 10.3389/fnut.2022.950338 36159483 PMC9493032

[12] Huang R Lin Y Ye X Zhong X Xie P Li M. Triglyceride-glucose index in the development of heart failure and left ventricular dysfunction: analysis of the ARIC study. Eur J Prev Cardiol (2022) 29(11):1531–41. doi: 10.1093/eurjpc/zwac058 35512245

[13] Huyett P Siegel N Bhattacharyya N. Prevalence of sleep disorders and association with mortality: results from the NHANES 2009-2010. Laryngoscope (2021) 131(3):686–9. doi: 10.1002/lary.28900 32681735

[14] Zhang X Sun Y Li Y Wang C Wang Y Dong M. Association between visceral adiposity index and heart failure: A cross-sectional study. Clin Cardiol (2023) 46(3):310–9. doi: 10.1002/clc.23976 PMC10018101 36651220

[15] Wu Z Tian T Ma W Gao W Song N. Higher urinary nitrate was associated with lower prevalence of congestive heart failure: results from NHANES. BMC Cardiovasc Disord (2020) 20(1):498. doi: 10.1186/s12872-020-01790-w 33238887 PMC7690024

[16] Liu Z Liu H Deng Q Sun C He W Zheng W. Association between dietary inflammatory index and heart failure: results from NHANES (1999-2018). Front Cardiovasc Med (2021) 8:702489. doi: 10.3389/fcvm.2021.702489 34307508 PMC8292138

[17] Su J Li Z Huang M Wang Y Yang T Ma M. Triglyceride glucose index for the detection of the severity of coronary artery disease in different glucose metabolic states in patients with coronary heart disease: a RCSCD-TCM study in China. Cardiovasc Diabetol (2022) 21(1):96. doi: 10.1186/s12933-022-01523-7 35668496 PMC9169264

[18] Wallace TM Levy JC Matthews DR. Use and abuse of HOMA modeling. Diabetes Care (2004) 27(6):1487–95. doi: 10.2337/diacare.27.6.1487 15161807

[19] Pan D Guo J Su Z Wang J Wu S Guo J. Association of the controlling nutritional status score with all-cause mortality and cancer mortality risk in patients with type 2 diabetes: NHANES 1999-2018. Diabetol Metab Syndr (2023) 15(1):175. doi: 10.1186/s13098-023-01138-2 37599357 PMC10440932

[20] Robin X Turck N Hainard A Tiberti N Lisacek F Sanchez JC. pROC: an open-source package for R and S+ to analyze and compare ROC curves. BMC Bioinf (2011) 12:77. doi: 10.1186/1471-2105-12-77 PMC3068975 21414208

[21] Khalaji A Behnoush AH Khanmohammadi S Ghanbari Mardasi K Sharifkashani S Sahebkar A. Triglyceride-glucose index and heart failure: a systematic review and meta-analysis. Cardiovasc Diabetol (2023) 22(1):244. doi: 10.1186/s12933-023-01973-7 37679763 PMC10486123

[22] Lopaschuk GD Karwi QG Tian R Wende AR Abel ED. Cardiac energy metabolism in heart failure. Circ Res (2021) 128(10):1487–513. doi: 10.1161/CIRCRESAHA.121.318241 PMC8136750 33983836

[23] Jia G Whaley-Connell A Sowers JR. Diabetic cardiomyopathy: a hyperglycaemia- and insulin-resistance-induced heart disease. Diabetologia (2018) 61(1):21–8. doi: 10.1007/s00125-017-4390-4 PMC5720913 28776083

[24] Henstridge DC Whitham M Febbraio MA. Chaperoning to the metabolic party: The emerging therapeutic role of heat-shock proteins in obesity and type 2 diabetes. Mol Metab (2014) 3(8):781–93. doi: 10.1016/j.molmet.2014.08.003 PMC4216407 25379403

[25] Alejandro EU Gregg B Blandino-Rosano M Cras-Méneur C Bernal-Mizrachi E. Natural history of β-cell adaptation and failure in type 2 diabetes. Mol Aspects Med (2015) 42:19–41. doi: 10.1016/j.mam.2014.12.002 25542976 PMC4404183

[26] Pan L Gao Y Han J Li L Wang M Peng H. Comparison of longitudinal changes in four surrogate insulin resistance indexes for incident T2DM in middle-aged and elderly Chinese. Front Public Health (2022) 10:1046223. doi: 10.3389/fpubh.2022.1046223 36530691 PMC9748338

[27] Li X Sun M Yang Y Yao N Yan S Wang L. Predictive effect of triglyceride glucose-related parameters, obesity indices, and lipid ratios for diabetes in a Chinese population: A prospective cohort study. Front Endocrinol (Lausanne) (2022) 13:862919. doi: 10.3389/fendo.2022.862919 35432185 PMC9007200

