# Supplementary material for: Association between the triglyceride glucose index and heart failure: NHANES 2007–2018
Source: Front Endocrinol (Lausanne). 2024 Jan 22;14:1322445. doi: 10.3389/fendo.2023.1322445 (PMC10839084; doi:10.3389/fendo.2023.1322445)
Supplement: Supplementary file 1 [file Image_1.pdf]

## Supplementary materials

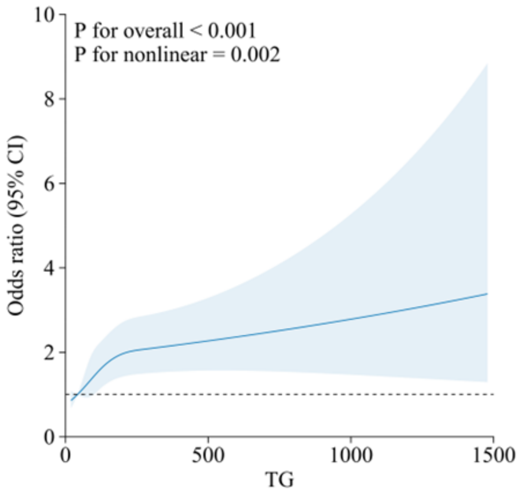


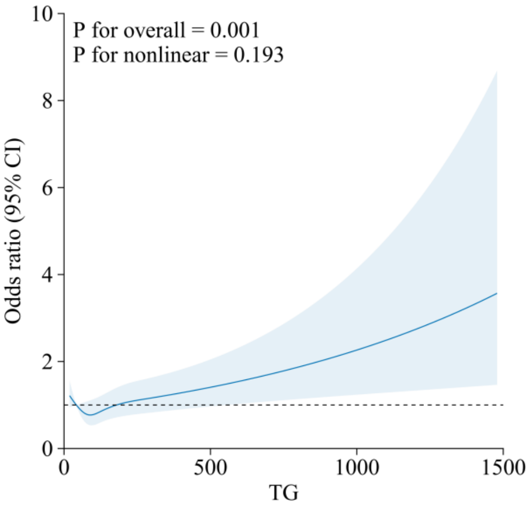


A B

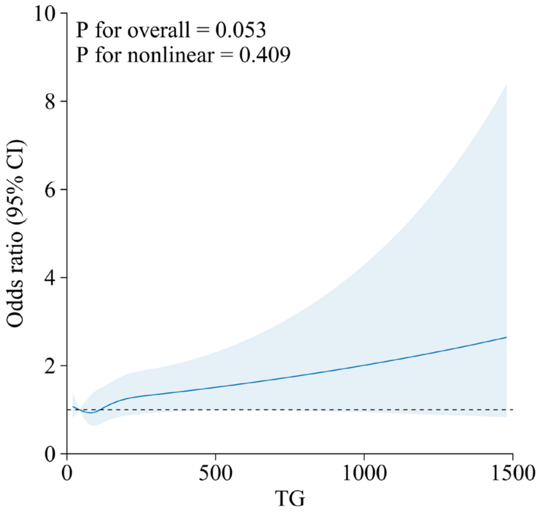


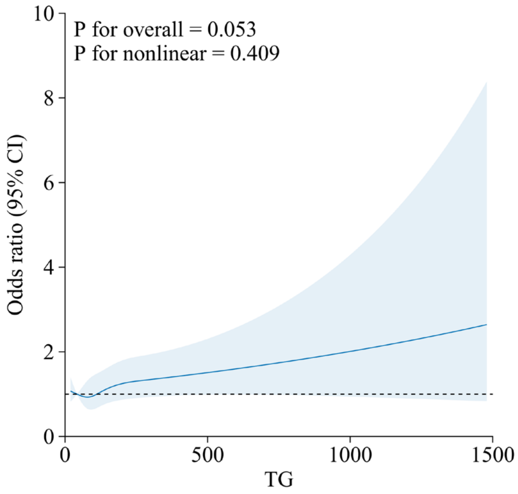


C D

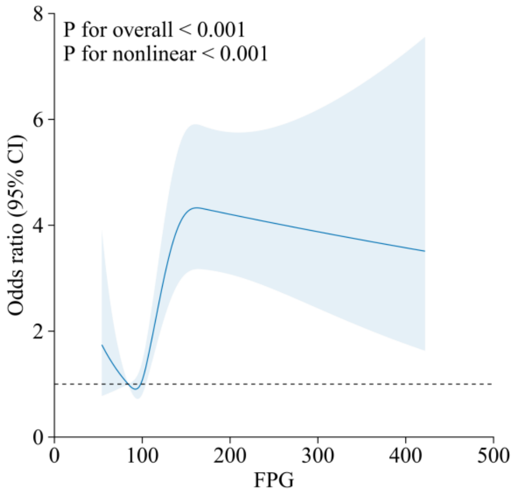


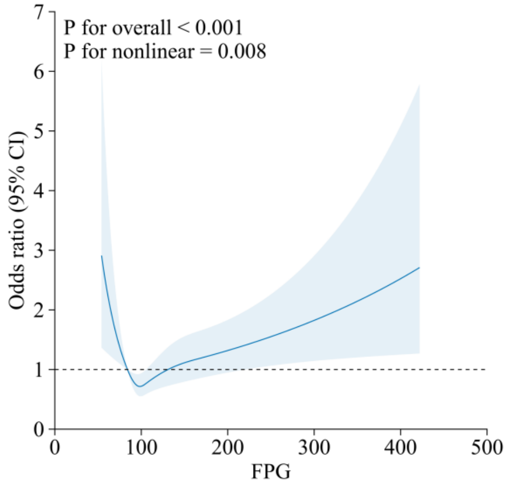


E F

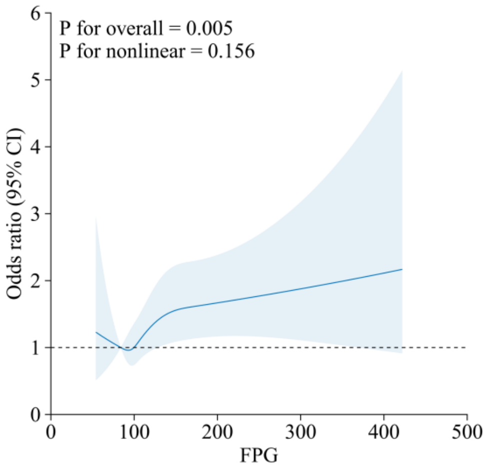


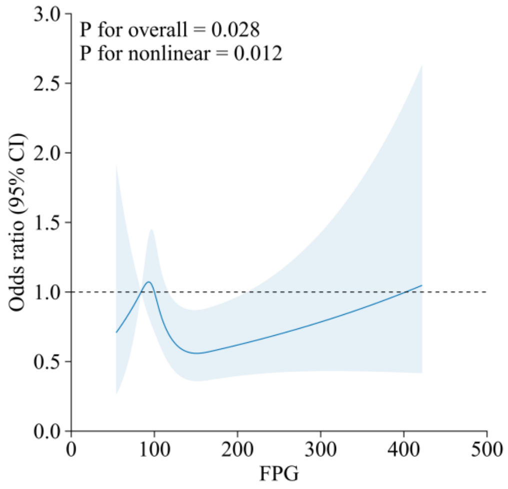


G H

**Supplementary Figure 1.** **A-D** and **E-H** represent Model1, Model2, Model 3 and Model 4. Model 1 was unadjusted. Model 2 was adjusted for age, sex, and BMI. Model 3 was adjusted for coronary artery disease, hypertension, heart attack, angina, and stroke. Model 4 was adjusted for age, sex, BMI, coronary artery disease, hypertension, heart attack, angina, stroke and diabetes. **A-D** showed the relationship between TG and HF risk. **E-H** showed the relationship between FPG and HF risk.
